# Evidence of interventions for improving healthcare access for lesbian, gay, bisexual and transgender people in South Africa: A scoping review

**DOI:** 10.4102/phcfm.v11i1.1367

**Published:** 2019-06-12

**Authors:** Zamasomi P. Luvuno, Gugu Mchunu, Busisiwe Ncama, Hlolisile Ngidi, Tivani Mashamba-Thompson

**Affiliations:** 1 Discipline of Nursing, School of Nursing and Public Health, University of KwaZulu-Natal, Durban, South Africa; 2 College of Health Sciences, University of KwaZulu-Natal, Durban, South Africa; 3 Discipline of Public Health Medicine, School of Nursing and Public Health, University of KwaZulu-Natal, Durban, South Africa

**Keywords:** lesbian, gay, bisexual, transgender, healthcare access, healthcare accessibility, homosexuality, LGBT guidelines

## Abstract

**Background:**

The lesbian, gay, bisexual and transgender (LGBT) populations have unique health risks including an increased risk of mental health problems, high usage of recreational drugs and alcohol, and high rates of infection with human immunodeficiency virus (HIV). Healthcare workers’ heteronormative attitudes compromise the quality of care to the LGBT population.

**Aim:**

The objective of this study was to provide an overview of documented evidence on South Africa interventions aimed at improving healthcare access for LGBT individuals using a systematic scoping review.

**Setting:**

This is a secondary literature review.

**Methods:**

An electronic search was conducted using the following databases: EBSCOhost, PubMed, Cumulative Index to Nursing and Allied Health Literature, and Google Scholar. Abstract and full article data were screened using inclusion and exclusion criteria by two researchers. Data extracted from the eligible studies were analysed using thematic analysis. The quality of the included studies was assessed using the Mixed Methods Appraisal Tool, version 2011.

**Results:**

Seventeen articles of the initial 151 hits were selected for review and an additional five files were identified through bibliographical search. Most studies had small sample sizes and focused on sexual health, targeting gay men and men who have sex with men in urban areas. Lesbians and bisexual women were not prioritised.

**Discussion:**

It emerged from the review that LGBT issues were not covered in the healthcare worker curriculum. Further it was noted that there is a paucity of data on the South African LGBT population, as sexual orientation does not form part of the routine data set. The findings of this review indicate gaps in the literature, practice guidelines and policies in LGBT healthcare in South Africa.

## Background

South Africa is the only African nation with legal assurances of equal rights for lesbian, gay, bisexual and transgender (LGBT) citizens, the same rights as their heterosexual counterparts. The South African Bill of Rights, Section 27,^1^ states that everyone has the right to access healthcare services and that no one may be refused services or treatment, or provided with inferior care, because of gender or sexual minority status.^1^ Despite institutionalised rights, evidence suggests that the LGBT populations encounter numerous structural and systemic barriers hindering access to quality healthcare.^2,3,4^ The reported structural barriers include scarcity of facilities that offer LGBT-targeted resources, and this is compounded by the lack of healthcare workers (HCWs) who are skilled in dealing with LGBT health issues.^5,6,7^ Systemic barriers include erasure of the LGBT populations in the healthcare system through the lack of utilisation data, practice guidelines and policies when dealing with LGBT issues. There is an assumption that LGBT populations do not exist in South Africa; hence, specific health data are not collected in healthcare facilities.^8,9^

The LGBT populations are very diverse but often grouped together.^10,11^ The sexual and gender minority population are clustered broadly in relation to sexual orientation and gender representation.^12^ Sexual orientation signifies the enduring pattern of sexual, romantic, physical and/or spiritual attraction. The terms *lesbian, gay, bisexual* and *transgender* are defined as follows: *Lesbian* denotes a woman attracted to other women; *gay* denotes a man who is attracted to men; *bisexual* denotes a person who experiences sexual, romantic, physical attraction to both men and women.^12,13^ *Transgender* is an umbrella term used widely to refer to a diverse group of individuals whose gender identity and expression diverge from culturally defined categories of gender, or denoting gender that does not conform to societal gender norms.^14,15^

Studies indicate that sexual and gender minorities face stigma, neglect and harassment in the hands of HCWs, who justify the poor treatment of the LGBT population using political, moral or religious beliefs to explain their behaviour.^16,17,18^ Consequently, the LGBT populations avoid healthcare facilities because they perceive health spaces as unsafe.^19^ In addition, this results in the LGBT populations experiencing increased risk of morbidity and mortality from preventable infections and cancers as they forgo health checks because of the hostility.^20^ Furthermore, the LGBT populations are reported to have an increased risk of alcohol and substance abuse, as well as mental health disorders, and are disproportionally affected by HIV infection.^21,22,23^ *South Africa’s National Strategic Plan on HIV, STIs and TB 2017-2022* recognises the LGBT populations as at high risk for acquiring HIV.^24^ The documented risks among the LGBT populations are not properly addressed nor managed.^16^ The main barriers contributing to this gap in quality care is that the HCWs are taught little or nothing about the unique aspects of LGBT health pre- and post-service.^6^ This is evident in the incompetency and lack of understanding of LGBT sexual health among the South African health workforce.^3,16^ Research on the South African LGBT population is sparse; the majority of research in LGBT populations is conducted in developed countries and thus does not align with the South African context.^8,19,25^ Finally, there is a lack of accredited courses on sexual health in South Africa.^6^

Currently, data on South African LGBT populations are limited.^26,27^ For example, sexual orientation and gender identity data are not collected in population surveys or the national census.^9,28^ The paucity of data on LGBT populations makes it difficult to plan evidence-based LGBT-targeted health programmes and to properly estimate the required resources for such programmes.^29^ This calls for introduction of an innovative, inclusive and respectful approach to collect data on sexual orientation and gender identity in the health care facilities. This will prevent misclassification, which can result in inappropriate medical care, and advise. Approaches such as inclusion of sexual orientation and gender identity information in the health surveys and census data to learn more about the demographics.^30^ Privacy may be maintained by using electronic data collection tools that will allow individuals to remain anonymous. The aim of this study is to map evidence of interventions for improving healthcare access for the LGBT populations in South Africa in order to identify areas for primary research and to help guide context-specific health policy development and practice guidelines for LGBT populations.

### Research question

Is there evidence relating to LGBT health interventions in South Africa?

### Objectives of the review

The objective of this review was to map the literature reporting on LGBT health interventions in South Africa.

## Research methods and design

### Identifying relevant studies

To identify studies relevant to the mapping of interventions that seek to improve LGBT health access in South Africa, we conducted a comprehensive literature search on articles published between 1996 and 2016. Various databases (EBSCOhost, PubMed, Google Scholar and the Cumulative Index to Nursing and Allied Health Literature [CINAHL]) were searched using the following key terms: *lesbian, gay, bisexual and transgender health*; *sexual and gender minorities*; *LGBT in South Africa*; *LGBT and health in South Africa*.

Two reviewers were responsible for the search and collectively designed the data-chronicling form, shaped according to the population, interventions, comparison and outcome (PICO) criteria. The search was limited to articles written in English and to studies based in South Africa. In the search, primary research studies, systematic reviews, letters and guidelines that address LGBT health issues in the South African context were included. The articles were then scanned for additional studies that were not identified by the search.

### Study selection

**FIGURE 1:**
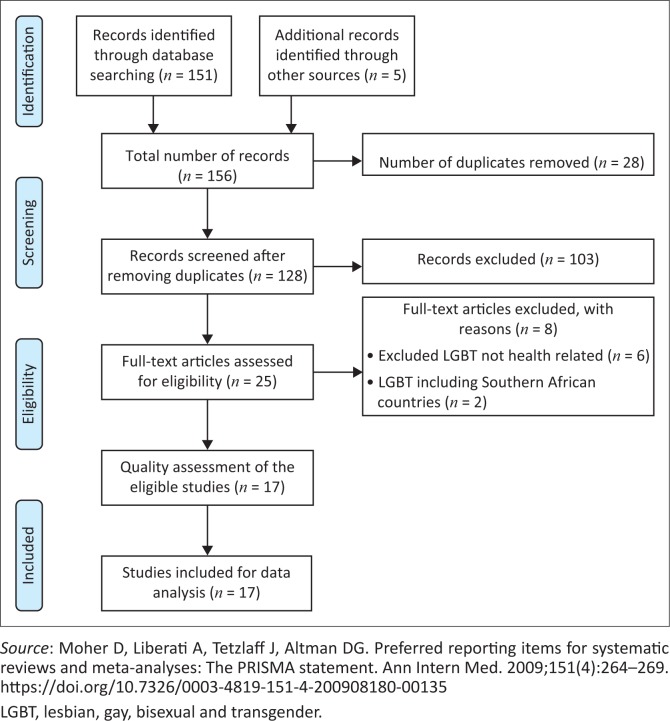
Preferred Reporting Items for Systematic Reviews and Meta-Analyses flow diagram showing phases of literature search.^32^

A comprehensive search of literature on the EBSCOhost, PubMed, Google Scholar and CINAHL databases was done based on our PICO inclusion and exclusion criteria. Literature search results were loaded to the EndNote (version 7) library. Two independent reviewers screened the titles and the abstracts against the inclusion criteria. All the titles and abstracts that met the inclusion criteria were selected, and full text reports were drawn. The reviewers screened the full reports for eligibility and meeting the inclusion criteria. The study selection process tracked the Preferred Reporting Items for Systematic Reviews and Meta-Analyses (PRISMA) flow diagram (see Figure 1).^32^

### Data extraction

The reviewers utilised a data-chronicling form to determine text words, variables and themes for inclusion and extraction to answer the research question (see Table 1).

**TABLE 1:** Summary of studies that were included in the scoping review.

Author and year	Study type and methods	Study population	Aims of the study	Key findings
Mprah (2016)^33^	Literature review guided by the UNAIDS Country Harmonization and Alignment Tool (CHAT)	-	Report on sexual and reproductive health needs of LGBT people in South Africa.	The South African constitution is all-encompassing and advocates for all citizens of the country, yet the LGBT populations are still stigmatised. The LGBT populations are not involved in the policy development. Data on LGBT sexual health issues is lacking in SA, as there are no surveys or data collected on the population. Most of the care and the services for sexual and reproductive healthcare are offered by NGOs because in government facilities no specialised care is offered. **Recommendations:** There should be large-scale research and surveys on LGBT sexual and reproductive health, with the full involvement of LGBT populations. It is important to train HCWs on LGBT issues.
Rispel and Metcalf (2009)^34^	Literature review and concept mapping using UNAIDS Country Harmonization and Alignment Tool (CHAT)	South African government policies on LGBTI government policymakers	Assess the extent to which the SA government policies and programmes cater for HIV in the MSM population; make recommendations for programme and policy implementation in the public health facilities.	SA HIV policies do not cater for MSM populations and have limited epidemiological and prevalence data on HIV in MSM populations. The South African government lacks stewardship in the care of LGBT populations. **Recommendations:** Policy research should be conducted to determine factors for inactivity on issues relating to HIV programmes and inertia in efforts to combat stigma and prejudice against LGBT populations in the SA public health sector. Include LGBT populations in HIV and AIDS surveillance in SA as part of the response. Engage the LGBTI populations as stakeholders in the development of policies and guidelines for the public health sector. Inclusion of sexual health services into the current health programmes and sensitivity training of HCWs pre- and in-service.
Newman-Valentine and Duma (2014)^35^	Literature review	Twenty-three studies reviewed on transsexual women from January 1972 to February 2013 (*n* = 172)	Exploration of health problems experienced by transsexual women, while navigating through a heteronormative health system.	Transsexual women are not catered for in legislation and health programme reforms. The health system is structured around cisgender males and females. Transsexual women have unique health risks, such as increased risk for cardiovascular problems because of lifelong feminising hormones. In addition, they are at an increased risk of HIV infection, mental health issues and increased suicidal tendencies. There is documented evidence of poor access to healthcare services, resulting in increased morbidity and mortality. Little is known about the transsexual population as data on the transsexual population is not collected in population statistics nor in the health system data; thus there is no evidence to motivate for care. **Recommendations:** Healthcare workers need to be trained on management of cross-gender hormones prescribed for transsexual women. Additionally, the health system should develop tools to collect data on transsexual individuals utilising health facilities.
Lane et al. (2008)^20^	Purposive sampling, in-depth interviews and focus group discussions (qualitative study)	Men who have sex with men (bisexual, gay and non-gay identifying MSM) (*n* = 50)	Describe experiences interactions of Gauteng MSM with public HCWs when seeking sexual health services.	MSM have limited access to non-stigmatising sexual health and experience homophobia and verbal abuse when accessing health services for STIs; as a result, they avoid discussing sexual behaviour with and/or deny same sexuality to HCWs. Gay-identified MSM and gender non-conforming MSM bear the brunt of HCW abuse, while non-gay identifying MSM are able to conceal their sexuality successfully, thus avoiding the homophobic outbursts of HCWs. **Recommendations:** Sensitise HCWs on LGBT population’s sexual health challenges and develop skills to prevent stigmatisation of the gender non-conforming MSM and the various MSM subgroups.
Smith (2015)^5^	Qualitative study tracking, cultural capital and intersectionality framework. Purposive sampling, semi-structured interviews (qualitative study)	Lesbian and bisexual women (*n* = 22)	Investigate healthcare experiences of lesbian and bisexual women in Cape Town, to understand how they experience healthcare, where and how they access sexual health information and the recommendations they may have for the healthcare facilities to be more inclusive.	Mixed results were found: women who accessed care at private facilities reported good interactions with HCWs, while those using government facilities were stigmatised and subjected to religious teachings. Homophobia or heteronormativity in the private sector was attributed to individual clinicians, while in the public sector it was attributed to the health system; all groups noted lack of sexual health information targeted at sexual minorities, healthcare facilities must provide more inclusive sexual health resources as this will improve the visibility of LGBT people in health facilities. Lack of HCW skills on sexual health also emerged. The participants suggested that HCWs should be trained on LGBT and comprehensive sexual health training and additionally have targeted patient education information and protective devices for safe sex. The women felt it would beneficial to have LGBT HCWs.
Muller (2013)^8^	Online survey to reach all lecturers who teach at the Health Sciences Faculty. Purposive sample (quantitative)	Academics (*n* = 93) focusing on LGBT content	To determine the extent to which LGBT health-related content is taught in the University of Cape Town medical curriculum through mapping and evaluation of LGBT teaching content in the Health Sciences Faculty in the Cape Town Medical School.	Only 10 academics, of the 93 who responded, taught LGBT-related topics for medical students. There was no structured curriculum to teach LGBT issues within all health disciplines. The knowledge, attitudes and practices of the medical students were not explored as part of the lessons. In disciplines such as the allied health professions and nursing, LGBT issues didn’t feature in their curriculum at all. It is of concern that even in the post-basic nursing curriculum, there was no content on LGBT health issues. Despite covering LGBT issues in the MBCHB curriculum, there was no formalised practical approach to assess the skills of students on LGBT issues. **Recommendations:** LGBT-related topics need to be incorporated into the health worker curriculum in order to equip students to provide competent care to LGBT patients.
Cloete et al. (2010)^36^	Convenient sample of PLWHA surveyed through self-administered questionnaire (quantitative)	HIV-positive women (641)	The study aimed to highlight the disregard of WSW in the South African HIV response, through a survey of PLWHA same-sex behaviour.	The results indicated that 11% (72) of the HIV-positive women surveyed reported sex with another woman, and 21 of the 72 indicated they were married to men. A proportion of the 72 women further indicated they engaged in both vaginal (44%) and anal (22%) sex without condoms. The above responses confirmed that although the women occasionally or regularly had sex with women, they also engaged in sex with men. Sexuality transmitted infections were reported by 76% (55) of the women. **Recommendations:** Research organisations should conduct large-scale research that can be used to influence modification in public policy on the responses and programmes to cater for WSW’s risks for HIV and AIDS.
Sandfort et al. (2008)^37^	Purposive sampling survey questionnaires administered either face to face or online (quantitative)	MSM population (*n* = 1045)	The study’s purpose was to investigate characteristics of MSM who tested for HIV and who tested positive.	There were 1075 respondents in the study, 87.8% of whom were attracted to other men and 12.2% who were attracted to both men and women. There was a high rate of testing in the sample (728 tested), with a high HIV prevalence of 14.1% in MSM who tested. Older, white and openly homosexual men were more likely to test for HIV. The data indicated that men who were students, compared to employed men, were likely to test. The men who tested HIV-positive were openly homosexual, suggesting encouragement to test within the LGBT population. **Recommendations:** Conduct a South African study on HIV prevalence in the MSM population and extract the results from the tested samples, thus basing the HIV status on self-reported information. National prevalence studies should be conducted employing inclusive sampling methods for MSM, such as respondent-driven sampling. Targeted studies on MSM sexual risk behaviours are recommended. It is important to be aware that MSM report bisexual behaviour, meaning HIV and AIDS general epidemic strategies need to include MSM as they are part of heterosexual partnerships.
Stoloff et al. (2013)^38^	Descriptive study with participants recruited from mental health clinic; data collection through structured clinical interviews (quantitative)	HIV-positive MSM (*n* = 25)	The study aims to describe psychopathology in HIV-positive MSM referred to the mental health clinic to inform the development of appropriate mental health services.	The results indicated that there were high rates of depression reported in nearly 50% of the sample, and 14 (56%) of the participants reported suicidal ideation. All participants screened positive for at least one personality disorder, with 80% screening positive for narcissism, 48% identified as having alcohol use disorder and 56% with drug use disorder. **Recommendations:** A multidisciplinary team should offer collaborative care in a MSM HIV clinic, with mental healthcare forming part of the HIV clinic. Mental health disorders are common in people living with HIV and AIDS, and poor mental health results in poor adherence to medication and risky sexual behaviour.
Muller (2014)^16^	Editorial	-	Motivation for provision of professional care for the LGBT population at healthcare facilities.	The LGBT population is discriminated against, ridiculed and subjected to personal religious beliefs by HCWs. Healthcare workers should be skilled and trained on LGBT patients and their specific needs. Muller argues that attitudes, knowledge and skills are linked. Lack of training of HCWs, he argues, is a barrier to access for the LGBT population because of the prejudice and discrimination the HCWs exhibit towards the LGBT population. The LGBT population perceives the health facilities as unsafe spaces; thus they avoid them and delay seeking care. Muller states that the negative attitudes exhibited towards LGBT patients are a result of the HCWs not being trained in LGBT health issues to allow them to challenge and question their attitudes towards the sexual minority population. **Recommendations:** Educate health professionals on the LGBT population and their health needs. The training needs to be conducted during basic training and in-service to improve HCW skills, improve their attitudes towards LGBT patients and thus improve access. Healthcare workers need to be reminded of their ethical and professional obligations in caring for LGBT patients.
Bateman (2011)^39^	-	-	Pleading a case for specialised healthcare services for people who identify as transgender in South Africa.	South African transgender people who aspire to transition are in a predicament, as the public sector has limited facilities that offer transgender services. Transgender services entail psychological assessment, hormone therapy and gender transition surgery. In South Africa, there are two public sector transgender clinics, the Steve Biko Academic Hospital in Pretoria and GSH in Cape Town, both supported by referral NGOs, the Triangle Project and Gender DynamiX. Bateman argues that SA transgender people have poor access to healthcare, as HCWs are not adequately prepared for management of transgender patients; this problem is further compounded by the stigma and prejudice towards transgender people. **Recommendations:** The health sector needs to demystify and destigmatise transgender people and get more people involved through educating the medical fraternity about sexuality. If HCWs are uncomfortable in treating transgender people, they must refer them.
Wilson et al. (2014)^7^	-	N/A	Outlined the challenges in the care of transgender people in South Africa with a focus on GSH Transgender Unit.	A transgender unit in GSH was established in 2009. The unit offers a comprehensive transgender care package, including hormone replacement therapy and gender-affirming surgery, and facilitates follow-up support in the local population. Limited funding and resources are the challenges currently facing the Transgender Unit, resulting in waiting times of up to 15–20 years for operations. Wilson et al. argue that transgender people’s poor access to services is compounded by poverty and lack of information. The limited number of HCWs trained on transgender care contributes to the problem. **Recommendations:** Innovation is required to increase local surgeons’ skills on gender-affirming surgery, by training them abroad. Transgender issues must be included in the HCW undergraduate curriculum, and a CPD-accredited course in transgender health must be developed for in-service education. Increase South African transgender research, as well as advocacy efforts to improve access and engage health policymakers to provide more inclusions for transgender policies. Revision of International Classification of Disease, 11th Revision (ICD-11) and Diagnostic and Statistical Manual of Mental Disorders, 5th Edition (DSM-5) is required to prevent transgender people being labelled as mentally ill.
Muller (2014)^40^	Editorial	-	A case and motivation for inclusion of the LGBT population in the training of HCW, as well as development of policies and programmes.	Gender identity and sexual orientation, like other social determinants of health, lead to health disparities and, compared with heterosexual and non-transgender socio-economically matched peers, the LGBT population is more likely to face barriers and experience stigma when accessing healthcare. There are gaps in the training of HCWs on LGBT health, and there is a high HIV prevalence in the LGBT population. It is necessary to introduce content relating to LGBT health issues in the HCW training, to allow HCWs to be able to do introspection, challenge their attitudes and develop skills on LGBT health issues. **Suggested course content:** Issues pertaining to LGBT health should be covered across the spectrum of HCW training – Urology, Infectious Diseases, Psychiatry, Public Health and Paediatrics. The training should include the following: history taking, development, risk factors and cancers associated with LGBT populations.
Imrie et al. (2013)^41^	Commentary	MSM in rural communities	The study illustrates that MSM in rural communities are not adequately studied in relation to HIV and the behaviours that drive HIV incidence. Authors argue that MSM in rural communities contribute to the incidence of HIV, yet they are understudied and not factored into HIV programmes.	The definition of sex in rural communities may be seen to mean only the sexual act meant for procreation; thus MSM sex may not be part of the definition of sex in that context. Men who have sex with men are not only disproportionately affected by HIV, but MSM behaviour contributes significantly to sustaining the high number of new infections recorded each year. No accurate estimates of South Africa’s MSM population exist, and only one national population survey has attempted to quantify their number. Because of a lack of understanding of MSM, particularly in the rural setting, they are not catered for in HIV programmes. Studies carried out on MSM are conducted in urban settings, and evidence indicates that access to healthcare is a challenge because of HCW discrimination; as a result, MSM delay seeking care. Authors argue that MSM in the rural population present as heterosexual in healthcare facilities to prevent being stigmatised. The authors emphasise that the MSM population is not homogenous and needs to be studied for a better understanding of what drives HIV in all settings. The authors state that in hyperendemic rural areas, higher numbers of lifetime partnerships carry an additional risk for HIV acquisition, which indicates that being linked into traditional culture makes self-acceptance, disclosure or discussion of same-sex behaviour or attraction with family, friends and healthcare providers very difficult. **Recommendations:** Conduct context-related studies on MSM as they are not homogenous. Interventions culturally appropriate to South Africa on MSM health programmes need developing to confer better delivery for these populations. Adapt evidence and interventions from both resource-rich and resource-limited settings, which may be carefully tapped into, tested and adapted for the South African health delivery platform and thus influence HIV programming and thus spread of good practice.
McAdams, Mahmoudet al. (2014)^42^	Purposive, snowball sampling with in-depth interviews (mixed method).	MSM (*n* = 90)	Examining minority stress and associated impact on mental health among MSM.	The MSM population faces stigma and discrimination both in society and places of care and service. The prejudice felt contributes to adverse mental health outcomes. The high-risk group, being young MSM with no family support, thus are at an increased risk of HIV infection and poor mental health outcomes.
Rispel et al. (2011)^43^	Key informant interviews, focus group discussion, and a survey (mixed method).	MSM and key informants skilled on MSM and HIV care (*n* = 202)	The study describes the availability and utilisation of HIV programmes and health services by MSM in South African cities in order to recommend improvements aligned to the NSP.	MSM find healthcare facilities to be unresponsive and associated with stigma and discrimination. Only 7% of the 152 participants were willing to choose government facilities for care; 96.1% would be willing to attend specialised MSM healthcare services and were interested in targeted messaging on safer sex for MSM, showing service acceptance. In contrast, 62.3% of the participants indicated they would prefer to access care at gay centres as opposed to heteronormative health facilities. **Recommendations:** Improve understanding of MSM needs–specific targeted programmes. Healthcare workers need sensitivity training to offer better care and learn lessons from the NGOs offering care to LGBT populations.
Stevens (2012)^4^	Snowball sampling followed by in-depth interviews (grey literature: either unpublished or published in non-commercial form. Examples of grey literature include government reports, policy statements and issue papers).	Gender non-conforming individuals (*n* = 80)	The study aimed to learn about the sexual health and practices of transgender people with the purpose of informing the development of new interventions or the adaption of existing evidence-based interventions to meet the unique HIV prevention needs of transgender populations.	Transgender people face barriers because of stigma, discrimination, abuse and unprofessional behaviour of HCWs when accessing healthcare services in SA public health facilities. Participants noted lack of skills and knowledge of HCWs in relation to LGBT health needs. The participants indicated that HIV risk factor health messaging was not relevant to their needs and noted a scarcity of safer sex protective devices (condoms, lubricant and pre-exposure prophylaxis) in state facilities. The transgender participants tabled various sexual practices with partners, including transactional sex and sex under the influence of alcohol and drugs. It also emerged that the transgender participants may be bisexual, homosexual or heterosexual. **Recommendations:** Healthcare workers should be trained on sensitivity towards the transgender population to be able to provide affirming health services. Further, the informational materials provided by HCWs in health facilities needs to be adjusted to cater for all individuals and take into account diversity of gender presentation; HCWs must be sensitive and use appropriate pronouns to demonstrate respect.

Note: Please see the full reference list of the article for more information.

LGBT, lesbian, gay, bisexual and transgender; SA, South Africa(n); HCW, healthcare worker; LGBTI, Lesbian, gay, bisexual, transgender and intersexed; MSM, men who have sex with men; MBCHB, Bachelor of Medicine, Bachelor of Surgery ; PLWHA, people living with HIV and AIDS; WSW, women who have sex with women; GSH, Groote Schuur Hospital; CPD, Continuing Professional Development programme; NSP, National Strategic Plan for HIV, TB and STIs; TB, tuberculosis; STI, sexually transmitted infection.

#### Inclusion criteria

- English language literature published between 01 January 1996 and 30 June 2016
- South African studies on LGBT physical health issues
- Lesbian, gay, bisexual and transgender state health interventions
- Full reports including qualitative, quantitative and mixed method studies.

#### Exclusion

- Studies conducted and published before 1996
- Studies not written in English
- Studies performed or reporting on LGBT issues outside South Africa
- Private health interventions
- Studies reporting on LGBT issues not physical health-related.

#### Keyword search

Boolean expressions (including *and/or*) were employed in combinations of the following keywords and phrases: *lesbian, gay, bisexual and transgender health*; *sexual and gender minorities*; *LGBT in South Africa*; *LGBT and health in South Africa*.

### Data collating, synthesis and summarising results

Six steps were undertaken to enable data collating and synthesis and to summarise the results of the scoping review. Firstly, researchers carried out thematic content analysis of the included studies using NVivo. Secondly, the two researchers independently coded the studies on LGBT people to find evidence of themes in literature on LGBT health. Thirdly, once the coding was complete the researchers met to synthesise the results and the codes, linking them to the scoping review objectives. Fourthly, data chartering was conducted, which involved documenting demographic data about the studies, the authors, study methods and design, including the number of participants and study setting. Fifthly, coded articles were themed on the topics and the focus covered in relation to the interventions relating to healthcare access for the LGBT populations. Finally, researchers met to collectively interrogate the studies and review how the identified themes linked to the aim of the review.

## Ethical considerations

Ethical clearance was not required as this study used secondary desktop data.

## Results

The initial search resulted in 151 references, with an additional five files found through scanning references of the extracted studies. Figure 1 provides an overview of the search and selection process. Ultimately, 17 articles were selected for this review. Table 2 gives an overview of the articles and the key findings using the following headings: author and year of publication; study methods and design; study population; aims of the study; key findings including recommendations. Of the 17 references, three were systematic reviews,^33,34,35^ two were qualitative studies,^5,20^ three studies used quantitative methodology^8,37,38^ and two triangulated the results using mixed methods.^42,43^ An additional five studies were identified that were editorials,^7,17,39,40,41^ and there was one grey literature article published on an organisation website.^4^ Most studies focused on sexual health (*n* = 6), with specific focus on men; there was only one study on women having sex with women (WSW) and one on transgender women. The sample sizes ranged from 22 to 1045. All the quantitative studies were based on respondent reports, and none of the studies were longitudinal.

**TABLE 2:** Population, intervention, comparison and outcome framework for the eligibility question.^31^

Criteria	Determinants
Population	The population of the study is LGBT people in South Africa who utilise state facilities for healthcare.
Intervention	Health interventions to improve physical healthcare access for the LGBT populations.
Comparison	None.
Outcome	Healthcare access guidelines for the LGBT populations in South African state health facilities.

*Source*: Peters M, Godfrey C, McInerney P, Soares C, Khalil H, Parker D. The Joanna Briggs Institute reviewers’ manual 2015: Methodology for JBI scoping reviews. South Australia: The University of Adelaide; 2015

LGBT, lesbian, gay, bisexual and transgender.

The Mixed Method Appraisal Tool (MMAT) was used for quality assessment of the included studies.^44,45^ In this study, all relevant studies were appraised in terms of methodology and quality using the MMAT checklists from appraisal outcomes, and motivation for decisions was kept for audit purposes. A second reviewer was asked to perform an independent appraisal of the selected studies.

### Analysis

Three main themes emerged from the scoping review: the South African terminology of LGBT, South African HCW skills, data and policies.

### South African terminology: Lesbian, gay, bisexual and transgender, men who have sex with men and women who have sex with women

Most of the studies (*n* = 7) did not categorise the participants as lesbian, gay or bisexual, according to sexual orientation, but rather described behaviour exhibited, that is, men having sex with men (MSM) and WSW.^20,36,37,41,42,43^ Men having sex with men is a social behaviour definition, including various identities of men who engage in sex with other men but do not necessarily self-identify as gay or homosexual, irrespective of the fact they have sex with women^34^; MSM population in the studies indicated they were attracted to both sexes. In a study by Sandfort and associates, 12.2% of 1045 participants indicated they were attracted to both men and women.^37^ Similarly, in the study by Cloete among 75 HIV-positive women, 21 of the women stated they had sex with women but were either married or in long-term heterosexual partnerships with men.^36^ Dlamini and associates argue that in the South African context, people who engage in same-sex relations do not necessarily identify as gay, as they will still have heterosexual relations, get married and have children in keeping with social norms.^46^ In South Africa, black people are not labelled according to sexual desires, as the expectation of procreation and marriage is paramount; same-sex sexual activity is ignored as long as there is marriage and reproduction to maintain the family name.^47^ Thus, the LGBT label may not essentially be appropriate in defining the South African MSM and WSW and gender non-conformity; rather the terms MSM and WSW are more suitable. The definition of these terms is important for HCWs to bear in mind when taking sexual history, to enquire about sexual behaviour and partnerships rather than the LGBT label.^4^ When a sexual history is taken from a transgender patient, sexual orientation should not be linked to the gender assigned at birth, nor gender identity, but must be confirmed with the particular transgender individual, to be able to assess the risk factors and offer relevant safer sex resources and messaging.^4,48^ Reproductive and contraceptive care must be part of the services offered to the transgender population, as there is a risk of unplanned pregnancy in transgender men engaging in unprotected receptive vaginal sex with men. Literature reports on unplanned pregnancies among transgender men, even while on exogenous testosterone.^49^

Absent from the studies are WSW and women who identify themselves as lesbians; little attention is paid to their HIV risk. The main focus is on MSM, yet there is evidence that WSW are also at risk of being infected with HIV and also face increased risk of mental illness, obesity and substance abuse.^36^ The National Strategic Plan (NSP) for HIV, tuberculosis (TB) and sexually transmitted infections (STIs) is also silent on WSW and women who identify as lesbian. The absence of WSW in the literature may be a result of societal homophobia and patriarchal views and an effort to correct same-sex behaviour in women.^30,36^

Four studies were on the transgender population; one study examined sexual health needs in grey literature, commissioned by a non-governmental organisation, which focused on the transgender population, and one literature review described the transgender woman.^4,7,35,39^ Two other documents found were editorials, highlighting lack of facilities for the transgender population. In the South African context, there are limited facilities to cater for the transgender population,^4,7^ which is subjected to stigma and discrimination not only in the public health sector but also in the private sector, as most medical aid companies exclude transgender care services and deem them cosmetic.^39^ International studies indicate that transgender women are at high risk of HIV, with a possibly similar situation in South Africa.^14,50^ Transgender individuals may have an increased risk of HIV infection because of a range of factors including poor access to employment, thus partaking in transactional sex.^14^ Further, transgender women may engage in unprotected receptive anal and oral sex, as this could be understood as affirming their female gender; conversely, this practice increases their risk of contracting HIV.^14,51^

### South African healthcare worker training on lesbian, gay, bisexual and transgender issues

Results indicate that South African HCWs are not equipped with training to deal with LGBT health issues preservice and in service^8,40^; as a result they lack sensitivity and exhibit various degrees of homophobia towards LGBT populations. This leads to the LGBT populations being isolated. Homophobia is defined as rejection, fear and irrational intolerance of same-sex individuals.^52^ The LGBT population reports being subjected to religious teachings, verbal abuse, micro-aggressions and sometimes being denied care by HCWs.^5,16,20^ The poor treatment of LGBT populations alienates them from accessing healthcare facilities, and they tend to attend health facilities only when complications have set in and it becomes an emergency.^4,17^ Nonetheless, if LGBT people do attend health facilities on a regular basis, it is highly possible that they do not disclose their sexual orientation; as a result they are treated as heterosexual, thus missing the opportunity to care for specific risk factors and related health screening.^20^

### Lesbian, gay, bisexual and transgender policies and guidelines for care in South Africa

The South African constitution has proclaimed the right to healthcare for all South African citizens.^1^ The South African healthcare system has not developed polices and practice guidelines to support LGBT people within the public health sector.^34^ Specialised relevant healthcare services that target the needs of LGBT populations are limited; available resources are constrained, limiting health access to the LGBT populations.^5,6^ Data on the LGBT utilisation of health facilities is limited, because data is not collected as part of the Department of Health data set, nor is it collected as population data.^9^ Thus prevalence of HIV among the South African LGBT populations is not documented, because sexual orientation data is not collected on large-scale, population-based HIV prevalence surveys and censuses.^35^ The few studies conducted on LGBT populations in South Africa have small sample sizes and were conducted in urban areas, disadvantaging the LGBT population in rural areas.^5,27,41^ The lack of data on South African LGBT populations means that designing programmes and developing related policy guidelines is a challenge.^29^ Documents such as the NSP for HIV, TB and STIs do identify the LGBT population as being at risk; an improvement is noted in the 2017–2022 plan, outlining supporting strategies to mitigate the risks identified.^53^

**FIGURE 2:**
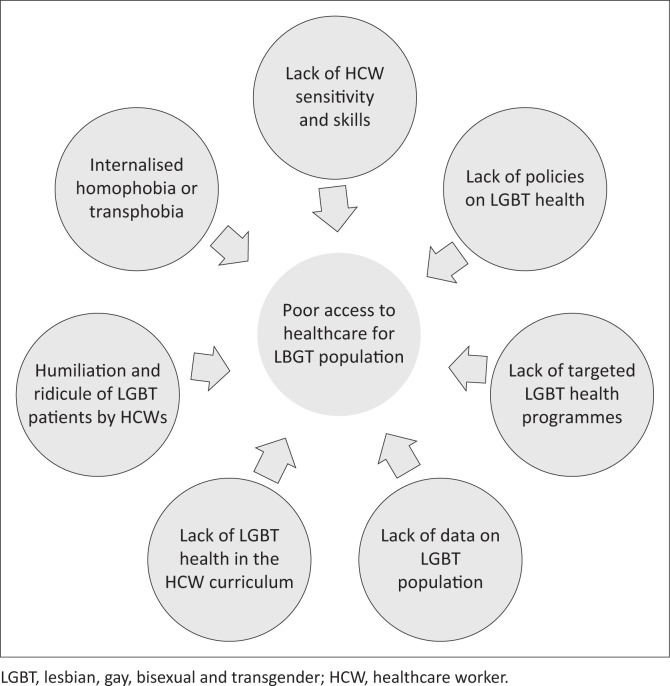
Diagram analysis of factors contributing to poor lesbian, gay, bisexual and transgender access to healthcare.

Figure 2 is a self-developed diagram representing the key themes. The LGBT populations have poor access to health services because of lack of information and skilled HCWs. Lack of data contributes to a paucity of policies, and stigma and discrimination by HCWs alienate LGBT individuals from health services.

## Discussion

The South African government has developed a constitution that is exceptional as far as human rights are concerned, granting rights to all South African citizens, including minority groups such as the LGBT population. However, implementation of the proclaimed rights has been found wanting.

Interventions are required, such as education of HCWs on LGBT care at an early stage of their careers and reinforcement at regular stages, to prepare HCWs to treat patients with professionalism and in a non-judgmental way.^8,40^ Further, healthcare interventions must focus not only on the diagnosis and treatment of illness but also on health promotion to empower LGBT clients to become advocates for their healthcare.^11^ Evidence indicates that HCWs are not well trained to deal with LGBT health issues, resulting in a lack of professionalism, demonstrated in poor attitudes towards the LGBT populations. In a study conducted in Kenya, a multidisciplinary team of HCWs was taken through 2-day sensitisation training on LGBT health issues followed by group discussion. Pretraining evaluation was conducted on knowledge and attitudes towards the MSM population. Post-training evaluation was carried out after 3 months and results reported improved knowledge of LGBT issues, correlated with reduced homophobic scores.^55^ This is an indication that improved health knowledge of HCWs on LGBT issues is likely to improve attitudes towards the LGBT population. Once HCWs are skilled on LGBT health issues, they are likely to conduct research and develop evidence-based care related to the South African LGBT population.^16,54^ It is apparent that there is a need to develop LGBT terminology relevant to the South African context and settings in view of the cultural nuances, inclusive of the rural population. The appropriate terminology will facilitate proper history taking and allow development of relevant sexual health messaging.

The LGBT populations are grouped in this scoping review, yet it is a diverse population with specific health needs and risk factors. It is important to study each subgroup individually.^29,55^ The scoping review indicates a paucity of information and interventions for WSW and transgender populations. More studies are required to understand the risk of WSW in acquiring HIV; this group needs focus in research, as their exclusion in HIV research suggests they are not at risk of HIV.^5^ In worldwide studies the transgender population is identified as being at high risk of HIV infection and are duly mentioned in the NSP as part of the high-risk group.^24,51,53^

### Limitations of the study

The literature is limited to studies published on South African LGBT populations and in English; studies in other languages have been omitted. Studies that may report on health-related LGBT issues in search engines outside of the health field were excluded. Only studies on LGBT issues from South Africa were used, possibly missing relevant issues relating to the LGBT population.

## Conclusion

The results of this scoping review indicate there are limited interventions targeted at the LGBT population. Failure to include sexual and gender minority health as part of the South Africa HCW training curriculum contributes to the neglect of LGBT issues, thus unconsciously limiting the production and circulation of knowledge on the sexual and gender minority population, as HCWs are not socialised to the LGBT population as they enter the various health professions.^6,16^ Research has shown that early career exposure of HCWs to LGBT patients increases clinical confidence and results in better patient experiences.^18,56^ Another possibility is that the key opinion leaders and policymakers have deliberately suppressed the production of knowledge and discussion of LGBT issues within the health system, to endorse the hegemonic view that instils the notion that all patients are heterosexual and cisgender.^18^ This renders a subset of society invisible, unseen and unheard; if they do speak up, they are often disregarded or punished at a macro level through lack of data and provincial policies and at micro facility level through HCW and population micro-aggression.^20,34,57^ There is a pervasive societal argument that sexual and gender minority is un-African, thus silently punishing those who transgress this view and relegating them to the margins.^46,58^

It is documented that LGBT populations experience a high risk of HIV acquisition.^26,27,34,36,59,60,61^ Despite the known risks, there is limited data on LGBT health and health policies to guide care, and this creates difficulties for the development of programmes that target the specific LGBT populations, as well as monitoring and evaluation of such programmes particularly in the areas of HIV care and prevention. Furthermore, there is a need for research on LGBT health, with all the subgroups studied independently as their needs and risk factors are diverse.^55^ Additionally, a qualitative study on HCWs is recommended to gain understanding of what drives the observed behaviour and attitudes towards LGBT populations. Engagement of LGBT populations on their experiences, views and expectations from the health services would be valuable to guide practice.

It is important to include data on the sexual orientation and gender identity of patients who utilise the healthcare facilities; this will aid in planning and programme development, particularly for STI health programmes.
